# Next Generation Sequencing of Ancient DNA: Requirements, Strategies and Perspectives

**DOI:** 10.3390/genes1020227

**Published:** 2010-07-28

**Authors:** Michael Knapp, Michael Hofreiter

**Affiliations:** 1 Allan Wilson Centre for Molecular Ecology and Evolution, Department of Anatomy and Structural Biology, University of Otago, 270 Great King Street, 9016 Dunedin, New Zealand; 2 Department of Biology, The University of York, Wentworth Way, Heslington, YO10 5DD, York, UK; E-Mail: michi@palaeo.eu

**Keywords:** ancient DNA, barcoding, genome sequencing, hybridization capture, targeted sequencing

## Abstract

The invention of next-generation-sequencing has revolutionized almost all fields of genetics, but few have profited from it as much as the field of ancient DNA research. From its beginnings as an interesting but rather marginal discipline, ancient DNA research is now on its way into the centre of evolutionary biology. In less than a year from its invention next-generation-sequencing had increased the amount of DNA sequence data available from extinct organisms by several orders of magnitude. Ancient DNA research is now not only adding a temporal aspect to evolutionary studies and allowing for the observation of evolution in real time, it also provides important data to help understand the origins of our own species. Here we review progress that has been made in next-generation-sequencing of ancient DNA over the past five years and evaluate sequencing strategies and future directions.

## 1. Introduction

The first study reporting ancient DNA (aDNA) sequences in 1984, predating the invention of PCR, was on 221 base pairs (bp) of mitochondrial DNA (mtDNA) obtained from an approximately 140 years old quagga skin [1]. In the late 1980s the invention of PCR [2,3,4] provided a significant boost to aDNA studies, including a much increased time depth of up to several tens of thousands of years. Nevertheless, until recently, analyses of aDNA were mostly restricted to short DNA fragments, usually derived from the mitochondrial genome, which is present in living cells in far higher copy numbers than the nuclear genome. Recent improvements in PCR technology have helped overcome some of the problems restricting ancient DNA research to short mtDNA fragments, but no innovation has revolutionized aDNA research to the extent that the emergence of next-generation-sequencing (NGS) has done. The first of these technologies was described in 2005 [5] and was almost immediately implemented in aDNA research. Within a few month of the introduction of NGS Poinar *et al*. [6] published 13 million bp from the nuclear genome of the extinct woolly mammoth. When compared with the 27,000 bp of cave bear sequence [7] that represented the largest nuclear data set available from an extinct species in the pre-NGS era, the data set obtained by Poinar *et al*. represented a 480x increase. This development has so far peaked in the publication of low coverage draft nuclear genomes of the woolly mammoth (0.8-fold [8]) and the Neanderthal (1-fold [9]) and also a high-quality 20-fold coverage nuclear genome of a 4,500 year old Palaeo-Eskimo [10]. 

The second, somewhat more recent technological change that is starting to influence aDNA research to an increasing extent is the move from PCR to hybridization capture as a method for enriching for the desired target sequence. For modern DNA, hybridization capture has successfully been used to enrich for large parts of the genome, for example the complete exome [11]. It is now starting to replace PCR as the method of choice for targeting specific sequences in the sea of contaminating environmental DNA typical for most aDNA samples [12,13,14,15]. 

In this review, we provide an overview of the strengths and weaknesses of the various next generation sequencing strategies used to sequence aDNA and the ways hybridization capture can be used to enrich for the sequence of interest, even from multiple samples. Finally, we discuss which directions aDNA research may take in the future.

## 2. Technical issues

### 2.1. Sequencing instruments and features

The four commonly used instruments for massively parallel sequencing are Roche (454) GS FLX Titanium, Illumina (Solexa) Genome Analyzer IIe, Applied Biosystems SOLiD and Helicos BioSciences HeliScope. A good overview is available in Millar *et al.* [16], although capacity and read length have increased since the publication of this review. The Titanium update of the GS FLX now produces about 400-600 million base pairs (megabases – MB) per instrument run with read lengths of up to 400 bp while the Illumina Solexa Genome Analyzer IIe produces up to 48 gigabases (GB) with read length up to two times 100 bp in paired end reads. Ancient DNA is usually highly fragmented with average fragment lengths ranging from 51.3 bp for some Neanderthal DNA [12] to 142 and 164 bp, respectively, for DNA from permafrost mammoth hair for which the largest DNA fraction was gel-purified [8]. Thus both the 454 and the Illumina read length is sufficient for sequencing aDNA fragments across their full length. Both platforms provide the possibility to physically separate lanes on a sequencing plate, thereby allowing for sequencing of multiple libraries without the necessity of barcoding. While a 454 picotiter plate can be separated into up to 16 individual lanes, the Illumina genome analyzer allows for separation into up to eight lanes. No studies using either the SOLiD or the HeliScope platform for aDNA sequencing have so far been published; therefore we will not discuss them in this review. Given the rapid speed of innovation in the field of next-generation-sequencing, it does appear likely, though, that both the SOLiD and HeliScope platform will be utilized for aDNA sequencing in the near future.

### 2.2. Selection of instrument

Apart from availability, the choice of instrument is mainly influenced by the desired application and the quality of DNA used. 

The latest model of the 454 sequencer provides average read lengths of more than 400 bp. This fragment length is sufficient to greatly reduce potential errors in *de novo* assemblies of consecutive sequence. However, of all next generation sequencing instruments it produces the smallest amount of data from a single run. This disadvantage is even greater if the average molecule length of the target is less than 400 bp, which is generally the case for aDNA, at least when the full range of aDNA fragments is targeted, be it via shot-gun sequencing or when using hybridization capture as enrichment technology. The reason for the on average relatively short fragment length of endogenous aDNA lies in the fact that the copy number of aDNA molecules increases by a factor of 2 -100 when the fragment length is divided by two [6,17]. However, when PCR is used to amplify relatively long fragments above 200 bp, which is often possible for permafrost samples (e.g. [18,19,20]), then 454 can be the most sensible choice. 

All other next generation sequencing instruments produce shorter read length but substantially more sequence data. Given the low amount of endogenous DNA and the short molecule length characteristic for aDNA, the larger amount of sequence data produced by these machines is a clear advantage for most aDNA studies. This is especially true for genome sequencing projects where the lower costs per nucleotide dramatically reduce both the overall price and the time required for a project [10], but also for studies using hybridization capture as usually the enriched sequences are not pure but are still embedded in a fairly high background of 60 – 80 % non-specific sequences [12,15]. 

### 2.3. Library preparation 

The preparation of sequencing libraries is very similar for both 454 and Illumina sequencing. First, molecules of ideal length for the respective sequencing approach are produced either by shearing longer molecules or by PCR amplification of ideally sized target fragments. As aDNA is characterized by strongly fragmented DNA molecules, shearing is usually not necessary, and may in fact be detrimental [21]. DNA sequencing libraries are in both cases constructed by ligation of universal adapters to both ends of target molecules. These sequencing adapters contain priming sites for sequencing and amplification. 

While both Illumina and 454 library preparation protocols are suitable for library preparation from modern DNA and other high copy number templates such as PCR products, they have limitations when dealing with low copy number aDNA extracts. The 454 library preparation protocol requires a streptavidin bead purification of adapter ligated template. A recent study [22] found that the last step, in which the non-biotinylated DNA strand of each molecule is released from the streptavidin capture beads by incubation with NaOH, is highly inefficient. In fact, only 0.1% of adaptor ligated library bound to the streptavidin beads was eluted from the beads using this approach. The authors found that replacing the NaOH elution step by short heat treatment increased the yield to 98%. 

The Illumina library preparation protocol is not ideal for aDNA either. The cohesive-end ligation of adapters described in the Illumina protocol requires one more purification step compared with the 454 blunt end ligation. It could be shown [22] that in each Qiagen Minelute purification between 16% and 40% of the template is lost, which makes each additional purification step detrimental with regard to the final yield of the library. Furthermore, the Illumina library preparation includes a pre-amplification gel purification step instead of the 454 streptavidin bead purification, which is not ideal for aDNA analyses. As well as being a potential source for the introduction of contamination, it may result in the loss of significant amounts of template.

Due to these shortcomings, new strategies to improve the efficiency and workflow of the library preparation process have been developed. Roche have recently introduced a rapid protocol to produce target fragments with adenine overhangs suitable for cohesive-end ligation of 454 sequencing adapters that does not require an extra purification step compared with the blunt ending procedure. Strategies for improving the efficiency of the Illumina libary preparation have also been developed [23]. These improvements in library preparation have dramatically reduced the amount of template DNA required. Therefore, for most applications, including shotgun sequencing even of less well preserved samples, library preparation is no longer a limiting factor in NGS analyses of aDNA. 

## 3. Next generation sequencing of ancient DNA

More than 20 studies have already made use of NGS to obtain sequence data from ancient remains (Table 1). Different sequencing strategies have been applied depending on the sample material and the objective of the study. 

### 3.1. Shotgun sequencing

Shotgun sequencing of aDNA can provide valuable information about the quality of the extract. It allows analyzing the composition of a DNA extract and to determine the relative abundance of endogenous DNA compared with contaminants. Ancient DNA extracts have been found to have very different levels of contamination. Sequencing 28 MB of DNA from a 28,000 year old, permafrost preserved mammoth from Siberia, Poinar *et al.* [6] found that at least 13 MB (45.4%) could be identified as being derived from the mammoth genome. Thus it takes only about twice as much sequencing effort to obtain the entire genome from this mammoth sample than it would to sequence for example a modern elephant genome. For sequencing the mammoth draft genome, a hair sample containing more than 90 % endogenous DNA was chosen [8] and a similarly high content of endogenous DNA was found for the hair tuft of a 4,500 year old Palaeo-Eskimo [10]. Preservation like this is however rare and can usually only be found in very young samples or in remains that have been preserved under optimal conditions such as permafrost. Subfossil remains of similar age preserved under temperate conditions such as Neanderthals and cave bears frequently contain less than 10% of endogenous DNA [7,9,24]. A further quality measure that can be determined from shotgun sequencing of aDNA is the average fragment length of the endogenous DNA. Fragment length hardly ever exceeds 400 bp and can be much shorter. Briggs *et al*. [12] found the average mitochondrial fragment length in five Neanderthal extracts to be between 51.3 and 79.3 bp. This information can be used to determine further sequencing strategies.

Shotgun sequencing represents the simplest sequencing strategy using NGS. It has been used for sequencing complete genomes of extinct species from well preserved permafrost mammoths [8,10] and from more poorly preserved Neanderthal remains [9,24] and sequencing of mitochondrial genomes from numerous species [36,37]. While a complete mammalian genome is about 2.8 - 4 billion bp, the mitochondrial genome is only about 17,000 bp long. Thus, the mitochondrial genome makes up only about 0.0004% of the entire genome. However, due to high copy numbers per cell, reads covering mitochondrial genome sequences are proportionally overrepresented among shotgun reads. In a recent study Gilbert *et al*. [25] found that, depending on the sample, between 0.08% and 1.99% of the reads obtained by shotgun sequencing mammoth extracts represented mitochondrial sequences. Thus, even the 454 sequencer, which produces only about 25,000 – 40,000 reads in its smallest run unit (1/16 of a full picotiter plate) will produce up to 800 mitochondrial reads. With a read length of 400 bp this would result in 18-fold coverage of the mitochondrial genome from each of the 16 lanes on a 454 picotiter plate, but even with an average read length of 100 bp the mitochondrial genome would still be covered 4-fold. 

However, this strategy is only suitable for very well preserved samples. Sequencing libraries from poorly preserved Neanderthal remains can contain less than 0.03% mitochondrial reads [28], as a much larger part of the overall reads is taken up by bacterial and other environmental DNA. The approach is also unsuitable for well preserved ancient samples if any other genomic region is targeted, as almost all other regions except for the mitochondrial genome are present in only 1-2 copies per cell. And even if the aim is to sequence mitochondrial genomes from well preserved samples, a success rate of 2% means that 98% of the reads are not useful for the analyses. Therefore, most aDNA studies require target enrichment strategies, which may soon also become the method of choice for complete genome sequencing, as they drastically reduce sequencing costs and have, in the form of hybridization capture, already been used to obtain high coverage DNA sequence data for ~14,000 protein coding positions of the nuclear genome from poorly preserved Neanderthal DNA [15]. 

### 3.2. Target enrichment strategies

Two main categories of target enrichment techniques commonly employed in aDNA research can be distinguished: 1) Polymerase chain reaction (PCR) amplification of target regions and 2) DNA capture via hybridization. 

**Table 1:** Ancient DNA studies using next generation sequencing partly or exclusively for sequence data collection.

Author/Date	Publication Title	Target region	Sequencing/ Target enrichment Strategy
Poinar *et al.* Jan. 2006 [6]	Metagenomics to paleogenomics: large-scale sequencing of mammoth DNA	genome	shotgun
Green *et al.* Nov. 2006 [24]	Analysis of one million base pairs of Neanderthal DNA	genome	shotgun
Gilbert *et al.* Sep 2007 [25]	Whole-genome shotgun sequencing of mitochondria from ancient hair shafts	mitochondrial genome	shotgun
Gilbert *et al.* Jun. 2008 [26]	Intraspecific phylogenetic analysis of Siberian woolly mammoths using complete mitochondrial genomes	mitochondrial genome	shotgun
Gilbert *et al.* Jun. 2008 [27]	Paleo-Eskimo mtDNA genome reveals matrilineal discontinuity in Greenland	mitochondrial genome	shotgun
Green *et al.* Aug. 2008 [28]	A complete Neandertal mitochondrial genome sequence determined by high-throughput sequencing	mitochondrial genome	shotgun
Miller *et al.* Nov. 2008 [8]	Sequencing the nuclear genome of the extinct woolly mammoth	genome	shotgun
Miller *et al.* Feb. 2009 [29]	The mitochondrial genome sequence of the Tasmanian tiger (Thylacinus cynocephalus)	mitochondrial genome	shotgun
Allentoft *et al.* Mar. 2009 [30]	Identification of microsatellites from an extinct moa species using high-throughput (454) sequence data	microsatellites	shotgun
Ramírez *et al.* May 2009 [31]	Paleogenomics in a temperate environment: shotgun sequencing from an extinct Mediterranean caprine	genome	shotgun
Willerslev *et al.* May 2009 [32]	Analysis of complete mitochondrial genomes from extinct and extant rhinoceroses reveals lack of phylogenetic resolution	mitochondrial genome	shotgun
Briggs *et al.* Jul. 2009 [12]	Targeted retrieval and analysis of five Neandertal mtDNA genomes	mitochondrial genome	capture
Zhao, Qi and Schuster Aug. 2009 [33]	Tracking the past: interspersed repeats in an extinct Afrotherian mammal, Mammuthus primigenius	genome	shotgun
Stiller *et al.* Oct. 2009 [34]	Direct multiplex sequencing (DMPS)--a novel method for targeted high-throughput sequencing of ancient and highly degraded DNA	mitochondrial genome	multiplex PCR
Krause *et al.* Feb. 2010 [13]	A complete mtDNA genome of an early modern human from Kostenki, Russia	mitochondrial genome	capture
Rasmussen *et al.* Feb. 2010 [10]	Ancient human genome sequence of an extinct Palaeo-Eskimo	genome	shotgun
Edwards *et al.* Feb. 2010 [35]	A complete mitochondrial genome sequence from a mesolithic wild aurochs (Bos primigenius)	mitochondrial genome	shotgun
Lindqvist *et al.* Mar. 2010 [36]	Complete mitochondrial genome of a Pleistocene jawbone unveils the origin of polar bear	mitochondrial genome	shotgun
Krause *et al.* Apr. 2010 [14]	The complete mitochondrial DNA genome of an unknown hominin from southern Siberia	mitochondrial genome	capture
Burbano *et al.* May 2010 [15]	Targeted investigation of the Neandertal genome by array-based sequence capture	nuclear genome	capture
Green *et al.* May 2010 [9]	A draft sequence of the Neandertal genome	genome	shotgun

#### 1) PCR

For both classical Sanger sequencing [38] and NGS approaches, PCR is still the most commonly used target enrichment strategy in aDNA research, although this situation may change in the near future. 

PCR target enrichment for NGS of modern DNA normally involves long range amplification of the target region followed by shearing of the PCR products to a size suitable for the NGS instrument used [39]. Ancient DNA however is usually highly degraded and not suitable for long range PCR. Therefore, if longer genomic regions such as the complete mitochondrial genome are to be sequenced, numerous independent PCR amplifications of overlapping short fragments are necessary. As aDNA extract is in many cases available in limited supply, it can be the limiting factor that determines how many PCR amplifications are possible. In 2005, a multiplex PCR approach that partially ameliorates this problem was introduced into ancient DNA research [20]. Instead of amplifying each amplicon separately, a number of non-overlapping amplicons are amplified in the same reaction, using the same amount of template as a singleplex PCR would have required. Using this approach, as little as two-non overlapping multiplex PCR may be sufficient to amplify long consecutive stretches of target DNA [20,40,41,42,43]. The PCR products from these first step multiplex amplifications can then be used in different ways.

In pre-NGS studies using multiplex PCR in connection with Sanger sequencing [20], the multiplex product was used as template for further second step singleplex amplifications. These second step amplifications were then used as template for Sanger sequencing. This approach can be adapted for NGS. All second step singleplex amplicons can be pooled, transformed into a sequencing library by ligation of the sequencing adapters required by the respective NGS instrument and then sequenced. The advantage of this approach is that singleplex amplicons can be quantified individually and pooled in equimolar ratios to obtain close to equal distribution of sequencing reads across all amplicons. The disadvantage is that singleplex amplification can be very time consuming if long target regions from highly degraded samples are to be amplified via a large number of very short amplicons. 

As an alternative to the singleplex amplification using the multiplex product as template, the multiplex template itself can be directly transformed into a sequencing library without further singleplex amplification [34]. This is a very time efficient approach, but it has the disadvantage that unequal efficiencies of different primer pairs in a multiplex PCR will manifest themselves in unequal sequencing coverage of different amplicons. This effect can be limited by optimizing cycle numbers in the multiplex PCR (enough to lift the target above the background but not too many to prevent too much variation in the copy number between poorly and well amplifying amplicons). Although such optimization requires additional sequencing and thus produces additional costs, this approach is to date the most time efficient PCR enrichment method for long target regions from degraded samples. 

Despite their status as the exclusive means of target enrichment in aDNA studies for about 20 years, PCR approaches have some significant shortcomings when used on aDNA. First and foremost, PCR requires a certain target molecule length to produce meaningful results. About 30 bp of informative sequence are needed to assign any given molecule confidently to its accurate coordinates within the genome [44]. Thus, if each PCR primer is 20 bp long, molecules of about 70 bp length are required to obtain 30 bp of informative sequence. As a consequence, many previous studies have targeted amplicons of 100 bp and more. However, 70 bp is already at the upper limit of average fragment length identified for many ancient extracts [7,10,12]. Even for permafrost preserved mammoth samples, average fragment lengths of more than 100 bp were only obtained by selecting the largest fragment fraction via gel electrophoresis [8]. Therefore, in almost all cases, PCR will only target a tiny fraction of the molecules at the very top end of the fragment length distribution, thereby greatly reducing information contained in the extract and increasing the risk of starting amplifications from single molecules. 

Especially in studies of humans and Neanderthals this effect can cause severe contamination of the resulting sequences. As modern human DNA can be an abundant source of contamination and modern human molecules will exceed 70 bp in length more often than the ancient molecules, all PCR approaches enrich for contaminating modern human molecules. Therefore, even small overall amounts of contamination can lead to erroneous sequences. In contrast, enrichment via hybridization capture such as PEC [12] targets the more abundant short endogenous aDNA molecules. This way, the ratio of endogenous to contaminating hominid DNA can be shifted substantially in favor of the former. This allows for authentic consensus sequences to be obtained even from samples that are relatively strongly contaminated when judged by PCR [13]. Finally, standard PCR (in contrast to quantitative PCR, which can however, not be applied to the multiplex approaches discussed above) does not reveal any information about the number of molecules a PCR started from. Therefore, at least two independent PCRs are required to ensure that not all reads covering any given site are amplicons of the same, potentially damaged or misread starting molecule [45]. Most of these problems of PCR discussed above do not occur in hybridization capture approaches. 

Nevertheless, it remains to be tested whether hybridization capture can outperform PCR enrichment if complex mixtures of species are targeted. In studies on sediment or ice core DNA [46,47,48], the use of conserved primers that flank variable regions has worked well. Given that hybridization capture relies on similarity between probes and target molecules, it may be difficult to develop capture systems that allow for the enrichment of variable regions across species. Probes targeting conserved regions adjacent to variable regions may represent a viable, albeit an as yet untested approach.    

#### 2) Hybridization capture 

**Figure 1:**
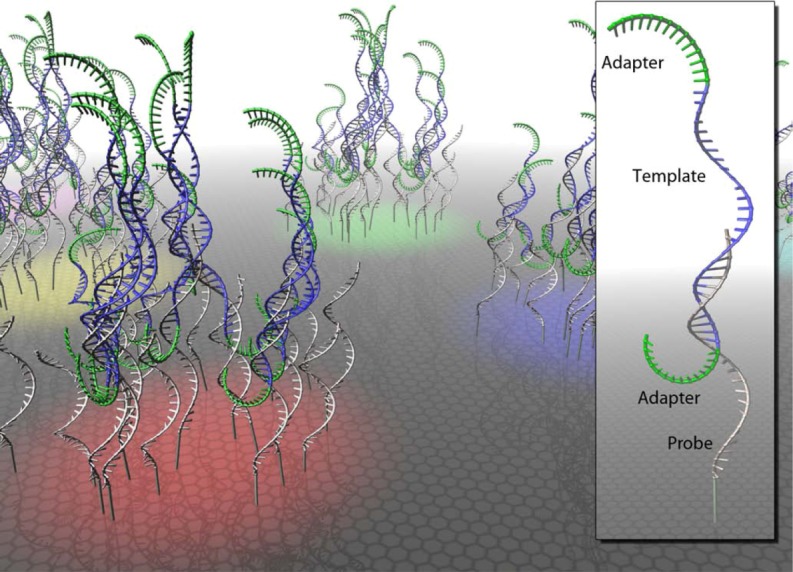
Hybridization capture on a microarray. Single-stranded oligonucleotide probes are arranged in clusters (red, yellow, green and blue surface colors) on a glass slide. Captured sequencing libraries consist of single-stranded target molecules (blue) and flanking sequencing adapters (green).

Numerous hybridization capture methods have been introduced for use with modern DNA. Strategies include on-array capture as well as in-solution capture [49,50,51,52,53]. In all cases target DNA is hybridized with specific probes that are either immobilized on microarrays (Figure 1) or can be immobilized on specific capture beads. 

So far, hybridization capture has been used in aDNA research to a very limited extent. The first study, to our knowledge, using a hybridization approach for targeting specific aDNA sequences was published in 2006 when Noonan *et al*. [54] showed that it was possible to capture specific, even nuclear, DNA targets from a batch culture of a Neanderthal library cloned into bacteria. They used biotinylated oligos that are, after an in-solution hybridization step, bound to streptavidin beads and targeted loci which they knew from sequencing several thousand clones from the bacterial library, should be present in the library. A different approach was taken by Anderung *et al*. [55], who used biotinylated oligos for capturing short fragments of mitochondrial DNA directly from aDNA extracts. This principle was taken up in 2009 by Briggs *et al*. [12,56] who used it to enrich mitochondrial DNA from five 454 Neanderthal sequencing libraries. 

In all cases, the principal is very similar to commercial in-solution capture strategies. Target specific, biotinylated oligos are hybridized to the genomic regions that are to be analyzed. The library/probe hybrid is then immobilized on magnetic, streptavidin covered beads. Non-target library is removed by washing and the target library is subsequently eluted from the streptavidin beads. The method is suitable for any NGS instrument, but it requires library preparation from aDNA extracts. Thus, the library preparation has to be optimized for very low copy numbers as described above. Moreover, this approach generally requires library amplification before hybridization to obtain a suitable amount of template and re-amplification of the enriched library after hybridization to ensure a sufficient DNA concentration for sequencing. 

Despite these additional amplification steps, hybridization capture as a target enrichment approach for aDNA overcomes some of the limitations of PCR enrichment. It requires a much shorter minimal fragment length than PCR and can therefore draw on a much bigger pool of endogenous, highly degraded molecules instead of just targeting the few long molecules from the upper limit of the fragment length distribution. This effect not only increases the potential to retain most of the original complexity of a sequencing library, it also reduces the risk of enriching modern contamination, as described above [13]. Finally, as individual molecules are sequenced and can be distinguished by their start and end coordinates with respect to the reference sequence, hybridization capture also allows determining the sequence coverage of each position in the same way as in shotgun sequencing. 

The main disadvantage of hybridization capture compared with PCR enrichment is the loss of template molecules during library preparation. It remains to be studied whether the potential to capture shorter molecules balances for this loss. In general it can be assumed that the shorter the average fragment length, the more efficient hybridization capture is compared to any PCR approach. 

### 3.3. Barcoding sequencing libraries

For many studies, for example in population genetics, even the smallest lane on any NGS instrument will produce excessive amounts of sequence data for a single sample. Moreover, if larger numbers of samples are to be analyzed, the costs soon become prohibitive if a full single lane is used per sample. It is therefore important to be able to pool multiple samples and sequence them in a single lane. As the information about sample origin of individual sequence reads is lost in all NGS approaches, barcoding techniques that put a specific tag on all DNA fragments from each respective sample and thereby allow reads to be assigned to samples using bioinformatics approaches are often necessary. Several different strategies are commonly used for barcoding sequencing libraries.

#### 1) Roche fusion primers

**Figure 2:**
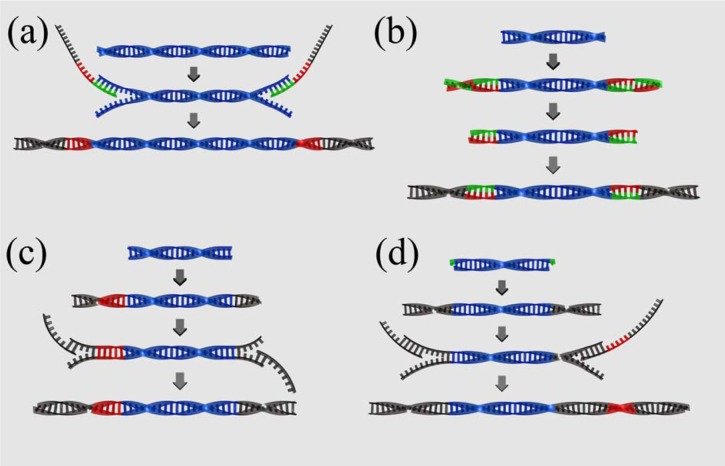
Comparison of barcoding strategies. **(a)** Fusion primers (454): Target molecules (blue) are amplified with primers consisting of target specific priming site (green), barcode (red) and 454 specific adapter sequence (grey). **(b)** Parallel tagged sequencing (PTS): Palindromic barcodes (red/green) are blunt end ligated to the ends of target molecules (blue). The outer half of the barcoding adapter (*i.e.*, the reverse complement part) is removed by restriction digest. Sequencing adapters (grey) are blunt end ligated to the ends of the barcoding adapters. **(c)** Direct multiplex sequencing (DMPS): One truncated, barcoded adapter (red and grey) and one truncated standard adapter (grey) are blunt end ligated to the ends of target molecules (blue). Adapters are extended to full length by PCR amplification with sequencing adapter specific primers. **(d)** Illumina barcoding (simplified): Truncated Illumina adapters (grey) are cohesive-end ligated to template molecules (blue) with adenine overhangs (green). Adapters are extended to full length by PCR amplification with sequencing adapter specific primers. One of the primers contains the barcoding sequence (red).

Roche offers a PCR based solution to transform PCR amplicons into barcoded or non-barcoded 454 sequencing libraries. Amplicons are amplified with oligos containing an amplicon specific priming site, an overhang containing the 454 adapter sequence and key and if desired a multiplex identifier (MID) between key and amplicon specific priming site (Figure 2 (a)). The approach is simple, quick and efficient, but it also has two major disadvantages. First, it requires an individual barcoded primer set for each individual that is included in the sequencing pool. This can rapidly increase costs and is therefore not feasible for population level studies. Second, the approach is unsuitable for library preparation directly from DNA extracts or sheared PCR products. 

#### 2) Parallel tagged sequencing

Parallel tagged sequencing (PTS) is a ligation based barcoding protocol [39,57]. In this approach, palindromic barcoding oligos are ligated to both ends of double-stranded target molecules to produce blunt ended, barcoded target molecules ready for library preparation (Figure 2 (b)). PTS can in principle be used with any NGS instrument. However, as the first sequenced nucleotide (i.e the first nucleotide 3' of the sequencing adapter) is identical for all molecules in this approach, it is not ideal for Illumina sequencing, as this will prevent the calling of sequence clusters. PTS is particularly suitable for the barcoding of any pre-amplified template. Templates can be barcoded individually and subsequently quantified to allow barcoded templates to be pooled in equimolar ratios prior to library preparation. However, similar to the fusion primer approach, the barcode is invariably located 3' of the sequencing primer and thus read as part of the sequencing read. It therefore effectively reduces the length of the target read by the length of the barcode (commonly about 7 – 10 nucleotides). While this is not an issue with the 400 bp reads of the 454 instrument, it is a limitation to be considered when the approach is used with instruments that produce shorter reads. A further disadvantage for aDNA studies is that the approach requires two subsequent adapter ligations. As each one is associated with a significant amount of template loss, the approach is not suitable for low copy number starting templates such as aDNA extracts or low cycle PCR products.

#### 3) Direct multiplex sequencing

Direct multiplex sequencing (DMPS) [34] was developed to facilitate barcoding of multiplex PCR enriched aDNA and allows for omitting the time consuming singleplex step. It follows very similar principles as PTS, but instead of blunt end ligating barcoding adapters independent from the standard library preparation adapters, DMPS allows for ligation of truncated, barcoded library preparation adapters followed by PCR amplification using primers that extend the adaptors to full length (Figure 2 (c)). It therefore requires one adapter ligation step less than PTS. In DMPS, multiplex PCRs are usually conducted with a minimum number of PCR cycles, to prevent well performing primer pairs in the multiplex set from outperforming less well performing primer pairs [34]. As a result of the small number of starting molecules in aDNA and the low number of PCR cycles, multiplex PCR products themselves usually have very small copy numbers, and can therefore not be barcoded efficiently using the comparatively inefficient PTS protocol.

DMPS can also be used to barcode any other double-stranded template. The main disadvantage of directly barcoding and sequencing multiplex PCR products is that the optimal PCR cycle number for the multiplex PCR is strongly depending on the respective sample and can only be determined by sequencing PCR products obtained using varying cycle numbers. Therefore, the optimization of direct multiplex sequencing is less cost efficient than sequencing pools of pre-quantified singleplex PCR products.

#### 4) Illumina barcoding

All the above barcoding strategies introduce barcoding sequences at the cost of target read length by adding barcodes 3' of the sequencing primer. Illumina uses a different strategy. Truncated universal sequencing adapters labeled P5 and P7 and including the sequencing primer priming site are ligated to DNA molecules. These adapters are then brought to full length by PCR amplification with tailed primers containing the sequencing primer priming site and a "crafting sequence" tail. The crafting sequence acts as the priming site for primers used to amplify single molecules in a bridge amplification during the sequencing process. In addition to the sequencing priming site and crafting sequence the P7 primer also contains the barcode in-between the sequencing sequence and the crafting sequence (Figure 2 (d)). Barcodes are read in an independent read starting from the P7 sequencing site. As a result, the target read is not affected by the barcode. A further advantage is that non-barcoded libraries with truncated universal adapters can be stored and barcoded independently for each experiment. Thus, unlike with all other barcoding strategies presented above, libraries are not assigned a fixed barcode, allowing for greater flexibility in experimental setup. 

## 4. Conclusion

Next-generation-sequencing of aDNA is associated with a number of problems and requires modification of existing protocols. Nevertheless, the large amounts of data produced by the various NGS instruments together with the short read length makes this new technology ideal for aDNA research. This is evidenced by the massive increase of available sequence data from long-dead organisms since the invention of NGS [58]. Due to the small amount of endogenous DNA and high background contamination characteristic for aDNA extracts, shotgun sequencing of aDNA is still only of limited use, and is mainly used for complete genome sequencing projects. Therefore, aDNA is likely to especially profit from new developments in barcoded and targeted sequencing. Capture methods in particular are a very promising approach. As the technology improves it is likely that even whole genome capture will be possible in the not too distant future, which will greatly reduce the costs and time for whole genome sequencing from extinct organisms. Next-generation-sequencing techniques carry the promise to bring aDNA research into the center of evolutionary biology and make it a crucial part of modern genetics. After all, what could help understand evolution better than following its progress in real time?
